# Diurnal rhythms of serum and plasma cytokine profiles in healthy elderly individuals assessed using membrane based multiplexed immunoassay

**DOI:** 10.1186/s12967-015-0477-1

**Published:** 2015-04-24

**Authors:** Raffaele Altara, Marco Manca, Kevin CM Hermans, Evangelos P Daskalopoulos, Hans-Peter Brunner-La Rocca, Rob JJ Hermans, Harry AJ Struijker-Boudier, Matthijs W Blankesteijn

**Affiliations:** Department of Pharmacology, Cardiovascular Research Institute Maastricht (CARIM), Maastricht University, 50 Universiteitssingel, 6229ER, P.O. Box 616, 6200MD Maastricht, The Netherlands; Experimental Vascular Pathology Group, Cardiovascular Research Institute Maastricht (CARIM), Maastricht University, Maastricht, The Netherlands; Department of Cardiology, Maastricht University Medical Center, Cardiovascular Research Institute Maastricht (CARIM), Maastricht, The Netherlands

**Keywords:** Cytokines, Multiplex immunoassay, Circulating biomarkers, Cardiovascular disease, Planar assay, Membrane-based assay

## Abstract

**Background:**

Recent clinical studies suggest that inflammatory mediators have huge potential in individualized therapy and in efficacy screening and can be utilized as biomarkers for a plethora of pathological conditions. The standard approach for detecting and measuring these inflammatory mediators is via blood samples. Nevertheless, there is no scientific report providing solid evidence on the most suitable blood compartment that will give the optimal inflammatory mediator measurement, or regarding the diurnal variation of circulating mediators. In this study, we present the biological variability of circulating cytokines and chemokines from healthy individuals (mean age 59 years) assessed by a novel membrane-based assay.

**Methods:**

Fifteen males and an equal number of females (all above 50 years) with no known inflammatory condition were selected. Through a planar method, named Proteome Profiler™, improved with fluorescence readout into a semi-quantitative multiplex assay, a screening of 36 inflammatory mediators was performed in serum and plasma of morning and afternoon blood withdrawals.

**Results:**

The multiplex analysis revealed that the physiological variability of several circulating inflammatory mediators was relatively small within a cohort of 30 healthy aging subjects. There was no substantial gender effect in the inflammatory mediator profile. On the contrary, most of the cytokine/chemokine values measured in the afternoon collection were found to be higher compared to the morning ones, particularly in plasma.

**Conclusions:**

In this study we provide evidence that circulating cytokine and chemokine levels of healthy individuals are elevated when blood is sampled in the afternoon compared to the morning, as influenced by the circulating cortisol levels. Furthermore, we report significant differences between cytokine/chemokine levels measured in serum and plasma. Our results provide essential information for future studies that will focus on examining circulating inflammatory mediator differences between healthy and diseased individuals.

## Background

The term “cytokine” was first introduced in the 70s [1]. The word “cytokines” entails hormone-like molecules secreted in response to inducing stimuli, which can originate from white blood cells or other sources (fibroblasts, endothelial cells, epithelial cells, etc.). In the last two decades the connotation of the word “cytokine” adopted various meanings, depending on the biological context where it has been identified. Starting from a mere role of effector in the cell-mediated immune response, cytokines have been shown to be involved in aging-related diseases where they orchestrate the physio-pathological development of several diseases [2,3]. Evidence from both experimental and clinical trials indicates that inflammatory mediators are of paramount importance in the pathogenesis of chronic heart failure (HF), contributing to cardiac remodelling and peripheral vascular disturbances [4]. Although the role of the cytokines during the progression to HF is still unclear, several lines of evidence suggest that inflammatory mediators play beneficial as well as detrimental roles in the development of the pathology [5] and can predict the development and the outcome of HF.

Chronobiology is a well-known entity [6] that plays a crucial role in many physiological and patho-physiological situations [7]. However, evidence about the diurnal variability of cytokines is scarce. Studies have focused on a limited panel of cytokines [8] leaving unexplored many others. However, profiling in time could add to clinical definition and management [9]. Furthermore, while sampling blood is a routine way for detecting and measuring inflammatory mediators, the selection of medium (blood compartment) can play a crucial role in the measured result (and is often imposed/dictated by the methodology and protocol offered by various assay manufacturers). Up to this point, no solid documentation exists on the medium that offers the most accurate results. In this study, we assessed the physiological differences of 36 circulating cytokines/chemokines in healthy aging male and female individuals, uncovering the inflammatory baseline for the aging inflammatory-related diseases. Furthermore, we evaluated the variability between morning/afternoon blood collection and the medium investigated: serum or plasma.

## Methods

### Subject population

Thirty healthy volunteers (15 males and 15 females) above 50 years of age were recruited under the supervision of a physician or cardiologist who overall judged whether each individual fulfilled the general criteria for a healthy person. The subjects were pre-screened and excluded in the case of an on-going chronic inflammatory disease, respiratory problems (asthma or COPD), hypertension, previous history of MI, recent illness of any kind in the last three months and/or use of anti-inflammatory drugs. Mean age ± SD of the volunteers was 59.4 ± 6.0 years (ranging between 50 and 71 years), and there was no gender-specific difference (average age for males 58.8 ± 6.0, ranging between 52 to 71 years and average age for females 60.1 ± 6.4, ranging between 50 and 70 years).

Volunteers were scheduled for the first blood withdrawal in the morning between 9-10 am and in the afternoon of the same day between 2-3 pm.

The recruitment was performed according to the Dutch Medical Ethical Committee (protocol: METC 11-3-056) and in accordance with the Declaration of Helsinki. All study participants signed the informed consent.

### Sample collection

Venous blood samples were collected from the cephalic vein (or antecubital vein) of the healthy volunteers. Serum and plasma were collected in special pre-vacuumed containers (Vacuette® K3 EDTA and Vacuette® plus clot activator, Greiner Bio-One, Austria). Blood was stored at 4°C for 3 h. Samples were then centrifuged at 2000 g for 10 minutes at 4°C and then immediately aliquoted and stored at -80°C until further analysis.

### Multiplex membrane-based immunoassay

Human Cytokine Array Panel A (R&D Systems™) was used according to the manufacturer instructions (see the Extracellular Factors session on www.rndsystems.com/product_detail_objectname_ProteomeProfilerArray.aspx) to determine the semi-quantitative presence of 36 cytokines. A total volume of 1000 μl (maximum possible volume) was poured per membrane for each individual and each condition that we were interested to determine (serum/plasma, AM/PM).

The aforementioned original Proteome Profiler protocol was adapted after step #9 in order to acquire semi-quantitative measurements by the use of a fluorescence read-out [10]. The analytes measured are expressed in Median Fluorescence Intensity (MFI) units.

### Statistical analysis

Raw data were imported in R. LIMMA package in R, a software environment for statistical computing; heat maps (http://www.r-project.org/) were used for hierarchical cluster analysis of the cytokines/chemokines in human blood. Graph pad statistical program (GraphPad Software, San Diego, USA) was used for graphics and statistical comparisons of data. Two-way ANOVA of repeated measures with a Bonferroni post hoc test was performed, in order to compare the difference between AM and PM measurements. Patients with missing values were excluded from the analysis. Values with *p* <0.05 were considered statistically significant.

## Results

In total, 36 inflammatory molecules were measured in blood samples of 30 healthy aging subjects. The complete sample set, i.e. plasma, serum, morning, afternoon withdrawal, was obtained from 22 subjects. Samples not included in the analysis did not differ from those included (data not shown).

**Figure 1:**
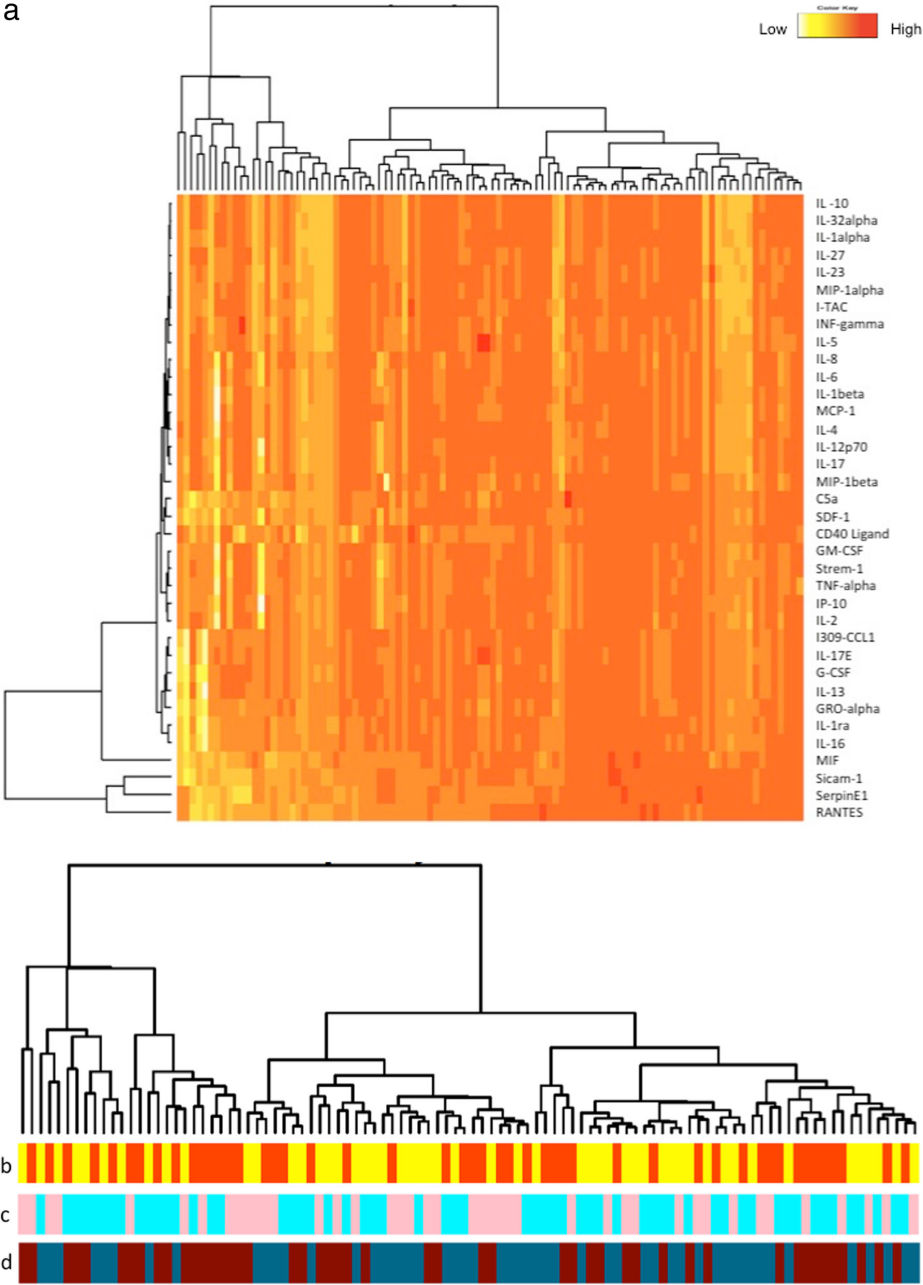
Two-dimensional cluster analysis of blood samples. **(a)** The similarity between individual profiles in different conditions clustered according to the dendrogram on the top, whereas, the relationship between inflammatory molecules by the dendrogram on the left. Sample times, genders, sample types distribution has been color coded and placed below the relative individual profiles cluster analysis (dendrogram on the top). The color codes are: yellow/morning, orange/afternoon **(b)**; blue/male, pink/female **(c)**; brown/serum, red/plasma **(d)**. The color-coded florescence intensity of all the values measured in the study are illustrated by the heatmap. White spots are missing values.

The use of fluorescence in the membrane assay approach [10] allowed us to observe which cytokines and chemokines were undetectable, absent or present in very low levels in serum or plasma. To better understand the complexity behind the inflammatory profiles in the different conditions a two-dimensional cluster analysis was performed (Figure 1a). Combining the sample clustering and the detected intensities of the inflammatory mediators, we generated three heat maps with the following conditions: gender difference, sample type and sampling time (Figure 1b, c, and d respectively). Although the aforementioned variables had a mixed and heterogeneous distribution, the differences in the pattern between female and male, morning and afternoon, serum and plasma, when graphically represented by the heat map were not observed: the clustering was based on the profile of the individuals, hence the output remained the same as in Figure 1a.

**Figure 2:**
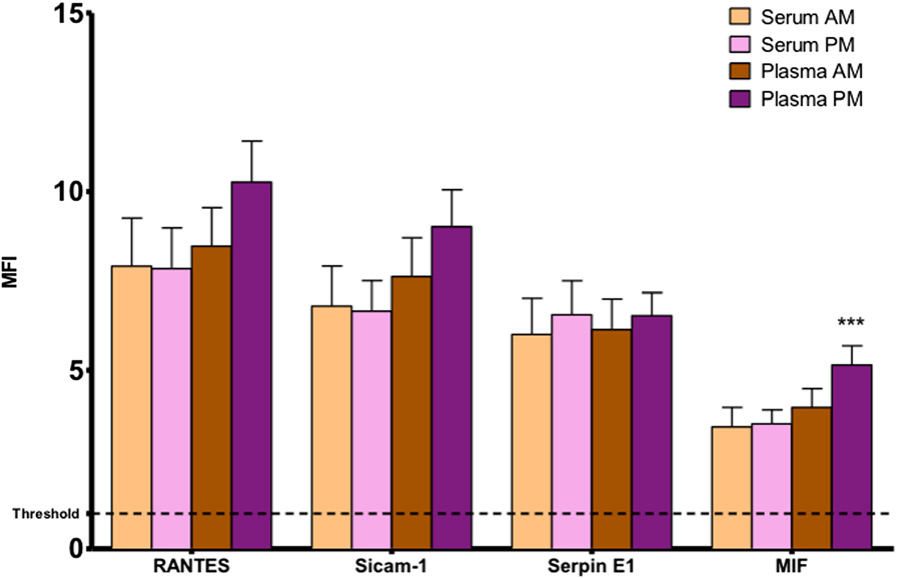
Relative expression of the four cytokines with the highest circulating levels. Four cytokines out of 36 assessed, were shown to have MFI above the threshold in every condition measured. Plasma PM has generally higher values compared to the ones measured in the other conditions (p < 0.001). ***p < 0.001 and corresponds to comparison to plasma AM. MFI, Median Fluorescence Intensity.

In order to determine which of the assessed analytes were the most abundant in the samples we chose a strict threshold. The reason for the use of the threshold was to make sure that the values obtained were sufficiently higher compared to the background. This cut-off point was set as twice the average of the values of the negative controls (MFI = 0.99). In Figure 2, four analytes - out of the 36 analyzed – are presented that were detectable with high intensities in all the conditions measured; these cytokines were RANTES (CCL5), Sicam-1, Serpin E1, MIF (see Table 1 for more details).

**Table 1:** **Serum and plasma levels of cytokines and chemokines for each condition measured**

		**Serum AM (n = 22)**	**Serum PM (n = 22)**	**Plasma AM (n = 22)**	**Plasma PM (n = 22)**
**High MFI**	**RANTES**	7.9 ± 1.4	7.8 ± 1.1	8.5 ± 1.1	10.3 ± 1.2
	**Sicam-1**	6.8 ± 1.1	6.7 ± 0.8	7.6 ± 1.1	9.0 ± 1.0
	**Serpin E1**	6.0 ± 1.0	6.6 ± 0.9	6.1 ± 0.9	6.5 ± 0.6
	**MIF** ^**††**^	3.4 ± 0.5	3.5 ± 0.4	4.0 ± 0.5	5.1 ± 0.5***
**Medium MFI**	**IL-16** ^**†††**^	1.2 ± 0.2	1.2 ± 0.1	1.2 ± 0.1	1.7 ± 0.1***
	**IL-1ra** ^**†††**^	1.2 ± 0.2	1.3 ± 0.1	1.2 ± 0.1	1.7 ± 0.1***
	**C5a** ^**†††**^	1.1 ± 0.1	1.1 ± 0.1	1.1 ± 0.1	1.6 ± 0.1***
	**GROa** ^**†††**^	1.1 ± 0.1	1.1 ± 0.1	1.0 ± 0.1	1.5 ± 0.1***
	**GM-CSF** ^**†††**^	0.9 ± 0.0	1.0 ± 0.1	1.0 ± 0.0	1.5 ± 0.1***
	**SDF-1** ^**†††**^	0.9 ± 0.1	1.0 ± 0.1	1.0 ± 0.0	1.5 ± 0.1***
	**CD40 Ligand** ^**†††**^	1.0 ± 0.1	1.1 ± 0.1	0.9 ± 0.0	1.3 ± 0.1***
**Low MFI**	**Strem-1** ^**††**^	0.9 ± 0.0	1.0 ± 0.1	1.0 ± 0.0	1.5 ± 0.1***
	**IP-10** ^**†††**^	0.8 ± 0.0	0.9 ± 0.1	0.9 ± 0.0	1.4 ± 0.1***
	**TNF-a** ^**††**^	0.9 ± 0.0	0.9 ± 0.1	0.9 ± 0.0	1.4 ± 0.1***
	**IL-2** ^**†††**^	0.8 ± 0.0	0.9 ± 0.1	0.9 ± 0.0	1.4 ± 0.1***
	**IL-8** ^**†††**^	0.8 ± 0.0	0.8 ± 0.0	0.9 ± 0.0	1.3 ± 0.1***
	**MIP-1ß** ^**††**^	0.8 ± 0.0	0.8 ± 0.0	1.0 ± 0.1	1.3 ± 0.0***
	**IL-1ß** ^**†††**^	0.8 ± 0.0	0.8 ± 0.0	0.9 ± 0.0	1.3 ± 0.0***
	**G-CSF** ^**†††**^	0.9 ± 0.1	0.9 ± 0.1	0.9 ± 0.1	1.4 ± 0.1***
	**I-309 /CCL1** ^**†††**^	0.9 ± 0.2	0.9 ± 0.1	0.9 ± 0.1	1.4 ± 0.1***
	**INF-g** ^**†††**^	0.7 ± 0.0	0.7 ± 0.0	0.8 ± 0.0	1.2 ± 0.0***
	**IL-1a** ^**†††**^	0.7 ± 0.0	0.7 ± 0.0	0.8 ± 0.0	1.2 ± 0.0***
	**IL-4** ^**†††**^	0.7 ± 0.0	0.8 ± 0.0^#^	0.8 ± 0.0	1.2 ± 0.0***
	**IL-5** ^**†††**^	0.7 ± 0.0	0.7 ± 0.0	0.8 ± 0.0	1.2 ± 0.0***
	**IL-6** ^**†††**^	0.8 ± 0.0	0.8 ± 0.0	0.9 ± 0.0	1.3 ± 0.0***
	**IL-10** ^**†††**^	0.7 ± 0.0	0.7 ± 0.0	0.8 ± 0.0	1.1 ± 0.0***
	**IL-12 p70** ^**†††**^	0.8 ± 0.0	0.8 ± 0.0	0.9 ± 0.0	1.3 ± 0.1***
	**IL-13** ^**†††**^	1.0 ± 0.2	0.9 ± 0.1	0.9 ± 0.1	1.3 ± 0.1***
	**IL-17** ^**†††**^	0.8 ± 0.0	0.8 ± 0.0	0.9 ± 0.0	1.3 ± 0.0***
	**IL-17E** ^**†††**^	0.9 ± 0.1	0.9 ± 0.1	0.9 ± 0.1	1.4 ± 0.1***
	**IL-23** ^**†††**^	0.7 ± 0.0	0.8 ± 0.0^##^	0.8 ± 0.0	1.2 ± 0.0***
	**IL-27** ^**†††**^	0.7 ± 0.0	0.7 ± 0.0^#^	0.8 ± 0.0	1.1 ± 0.0***
	**IL-32a** ^**†††**^	0.7 ± 0.0	0.7 ± 0.0	0.8 ± 0.0	1.1 ± 0.0***
	**I-TAC** ^**†††**^	0.7 ± 0.0	0.8 ± 0.0^#^	0.8 ± 0.0	1.2 ± 0.0***
	**MCP-1** ^**†††**^	0.7 ± 0.0	0.8 ± 0.0^#^	0.8 ± 0.0	1.2 ± 0.0***
	**MIP-1a** ^**†††**^	0.7 ± 0.0	0.7 ± 0.0^#^	0.8 ± 0.0	1.2 ± 0.0***

NOTE. ^#^significant at p < 0.05, ^##^or ^**††**^significant at p < 0.01, *** or ^**†††**^significant at p < 0.001.

MFI = Median Fluorescence Intensity.

The table is divided in three parts: the “high” intensities (on top) represent the cytokines that are more abundant in the circulation. The “medium” intensities (in the middle) represent the cytokines with MFI values above the threshold for at least two conditions. The “low” intensities (on the bottom) represent all the cytokines with MFI values below the threshold. Values are expressed in MFI ± SEM. Differences compared to “Serum AM” are marked with ^#^and the ones compared to “Plasma AM” with *. The statistical analysis with two-way ANOVA further demonstrated that there is a “significant interaction” between serum and plasma. Therefore, the increase between AM and PM values is dependent on the type of matrix (serum or plasma) that is used. This interaction is indicated by ^**†**^for each corresponding cytokine/chemokine that is found to show a “significant interaction”.

**Figure 3:**
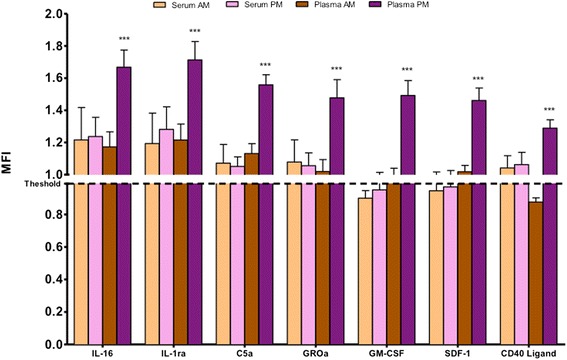
Relative expression levels of the analytes with circulating levels above threshold in (at least) two of the four conditions tested. The graph represents the selection of the analytes that showed low signals but were still above the threshold for at least two conditions. Note that all plasma PM values are significantly (*p* < 0.001) increased compared to Plasma AM. MFI, Median Fluorescence Intensity.

From the remaining 28 inflammatory mediators measured, 10 were found to have a fluorescent intensity above the threshold in at least two of the four conditions tested (Figure 3). Interestingly, with the exception of Serpin E1, we consistently observed higher levels in the plasma sample taken in the afternoon, compared to serum and morning plasma values (Table 1).

## Discussion

To our knowledge, this is the first study in which an extensive range of physiological cytokine and chemokine profiles is compared between healthy males and females. Furthermore, this study provides evidence for the essential variability that might occur when blood is sampled as plasma or serum and at two distant time points along the circadian rhythm (AM/PM).

Age-related changes in circulating inflammatory profiles might be the key point to focus on when implementing the diagnostics and prognostics of various diseases, i.e. cancer [11], HF [12] etc. Most importantly, there is scientific evidence that answer to drugs administration can be stereotyped, based on time of administration [13]. In the last two decades, there has been an abundance of reports on the involvement of the inflammatory response to the onset of HF [14], however a top selection of the inflammatory biomarkers has not yet been accomplished. With reference to seminal studies which report a variation between the HF incidences in men and women (women with HF are better protected against apoptotic death signals and show a later onset of cardiac decompensation compared to men) [15], we studied the physiological thresholds in both genders. Furthermore, this investigation was conducted within an aged population where the risks of HF development are known to be higher [16,17], and the circulating inflammatory mediators are expected to be elevated [18]. To achieve this, we generated hierarchy of clusters from the intensities detected in every individual for each condition measured (male/female, AM/PM collection, plasma/serum sampling). All observations started in one cluster (Figure 1a), and splits were indiscriminately generated as one moves down the hierarchy. The colored stripes originating from the cluster analysis clearly indicated that the physiological inflammatory profile is evenly distributed for every selected parameter (gender, sampling time, sample type). In other words, the clustering of both genders seen in our study (Figure 1b) is showing that no significant difference based on gender is present. These results suggest that the onset of an inflammatory-associated disease is not triggered by a difference in the normal physiological levels of the circulating cytokine profiles. This hypothesis should be further confirmed in a larger retrospective study with the inclusion of individuals that progress into HF development.

The choice for the sampling between serum or plasma is often driven by the requirements of the immunoassays that the investigator is planning to utilize and this is usually imposed by the specific assay manufacturer/supplier used. Choosing serum rather than plasma always raises the questions whether in the “other” sample the levels of the same analyte of interest would be different (and vice versa). In some studies it was demonstrated that a limited number of circulating cytokines have a different concentration outcome when measured in either serum or plasma [19]. Various scientific groups have demonstrated serum-plasma variability owing to a variety of factors, such as the release of cytokines from platelets during clotting or centrifugation [20,21], the type of blood collection tubes [22], degradation during the clotting process [21], as well as the “matrix effects” of multiplex systems and sample preparation artifacts (leading to non-systematic differences) [23]. We cannot exclude that one of these factors could have influenced the small differences observed. However, to pinpoint which of those factors is the real responsible, a different study focusing on the methodological aspects would be more appropriate.

Incidentally, when comparing serum *vs.* plasma in our measurements by membrane-based assay, a strong trend of high given values in “plasma PM” was observed. Indeed, for almost all the inflammatory mediators measured, the plasma PM values were significantly higher than in the other conditions (serum PM or serum AM). However, no profound interpretation of morning/afternoon changes could be given. This is indeed a limitation of the study, since we did not measure other potential factors that might have been responsible for the small variation observed. Unfortunately, this area in literature is still grey and no conclusive explanation has been given regarding the differences observed between measurements of inflammatory mediators in plasma or serum [21]. In addition, Wong et al. have shown that plasma cytokine values are consistently higher compared to the ones measured in serum [21]. Lastly, the de Jager group has reported important differences in the cytokine levels depending on the sample type. Serum samples show higher levels of cytokines at 4°C compared to plasma samples and this seems to be due to the fact that serum samples have generally higher levels of chemokines [22,24].

Furthermore, a large piece of evidence suggests that the diurnal rhythm has a pronounced effect on a variety of physiological or pathophysiological situations [7] - including the cardiovascular system [9]. In addition, recent studies such as the one from Benito et al. on teat cytokine/chemokine levels, have been pointing towards a time-dependent relationship as regards to the levels of several important analytes, such as IL-1β, IL-10, CXCL1, CLXL10/IP-10, VEGF etc [25]. However, to the best of our knowledge, no systematic effort has been conducted so far to systematically investigate the diurnal variation of chemokines and cytokines, and their potential impact on classification at screenings/general-practices. We hypothesized that measuring cytokine/chemokine levels in different time points of the diurnal rhythm would demonstrate variable inflammatory mediator levels. Indeed, we showed that the levels of almost all the analytes measured - with the exception of Serpin E1 and RANTES - were elevated in plasma PM samples when compared to the other conditions assessed. The increased levels of several inflammatory mediators during the PM blood collection point can be attributed to the effect of low cortisol-circulating levels. It is well-documented that human cortisol levels peak in the morning (approximately 8.30 am) and reach a minimum around 22.30 pm [26] and it has been shown that cortisol has a suppressive effect on IFN-gamma, TNF-alpha, IL-1alpha, IL-12 [8], IL-6, as well as inhibitory effects on Th1 and Th2 immune response [27]. On these grounds, high levels of circulating cortisol (peaking at AM sampling), lead to depressed levels of the cytokines under investigation. This hypothesis is in agreement with well-established knowledge from previous years, showing that glucocorticoids (and cortisol specifically) are potent inhibitors of the pro-inflammatory and inducers of the anti-inflammatory hormones in the body [28,29].

## Conclusions

Plasma collected in the afternoon contains higher concentrations of cytokines and chemokines than serum and plasma collected in the morning and serum sampled in the afternoon. Overall, the aim of this study was to expedite the transition of the circulating inflammatory markers, in order to investigate their potential implementation in the clinical setting as biomarkers. Collectively, the present data provide important information regarding the diurnal rhythm of cytokine/chemokine profile in serum and plasma in healthy aged individuals.

In conclusion, this study demonstrates the importance of diurnal rhythm in the regulation of a wide range of cytokines/chemokines and provides evidence for the first time for the variability of their levels depending on the medium (plasma or serum). Hence, great consideration should be given on the blood sampling time, as well as the use of serum or plasma, in order to avoid inconsistencies in the measuring of inflammatory mediators for prediction of HF or any other pathological condition. Furthermore, this proves the great need of a globally standardized method/protocol in measuring inflammatory cytokines/chemokines, in order to allow for the reproducibility and comparability between different research groups/laboratories. Introducing a unified method in sampling/measuring and defining the underlying reasons for the variabilities and their mechanisms could prove of crucial importance in the hunt for novel biomarkers in HF, cancer and any other pathophysiology characterized by an inflammatory response.
